# Root and Canal Morphology of Mandibular Third Molars in an Iranian Population

**DOI:** 10.5681/joddd.2012.018

**Published:** 2012-09-01

**Authors:** Maryam Kuzekanani, Jahangir Haghani, Hossein Nosrati

**Affiliations:** ^1^Oral & Dental Diseases Research Center, Kerman University of Medical Sciences, Kerman, Iran; ^2^Associate Professor, Department of Endodontics, Faculty of Dentistry, Kerman University of Medical Sciences, Kerman, Iran; ^3^Associate Professor, Department of Oral and Maxillofacial Radiology, Faculty of Dentistry, Kerman University of Medical Sciences, Kerman, Iran; ^4^Dentist, Private Practice, Kerman, Iran

**Keywords:** Anatomy, mandibular third molar, morphology, root canal

## Abstract

**Background and aims:**

A through knowledge of the root canal morphology is required for successful endodontic ther-apy. The aim of this study was to investigate the root and canal morphology of mandibular third molars in Kerman, a prov-ince in southeast of Iran.

**Materials and methods:**

One-hundred-fifty extracted mandibular third molars were collected randomly from different dental clinics in Kerman. The root canal anatomy and morphology of each tooth was carefully studied using a clearing tech-nique. Root number and morphology, number of canals per root, root canal configuration according to Vertucci classifica-tion, and incidence of dilacerated roots and C-shaped canals in mandibular third molars were evaluated under stereomicro-scope with ×2 to ×3 magnifications.

**Results:**

From the total of 150 mandibular third molars studied, 21% had one root. The majority of teeth (73%) had two roots. 5.5% of the teeth had three roots. The incidence of C-shaped canal was 3.5% in this study and 8% of the teeth had at least one dilacerated root.

**Conclusion:**

Although root canal anatomy and morphology of mandibular third molars is very variable having two roots seems to be the normal anatomy for these teeth.

## Introduction

Loss of the mandibular first and second molars is often the reason the third molar must be considered as a strategic abutment. Another indication for root canal therapy and full coverage is a fully functioning mandibular third molar in an arch that has sufficient room for full eruption and oral hygiene. The anatomy of third molars has been described as unpredictable.^1^

Nevertheless, the prerequisite for restorative and prosthetic treatments of these teeth is to perform a successful endodontic treatment. To this end, knowledge of the internal anatomy of the teeth is critical.^2 - 4^ Since radiographic images only provide a two-dimensional evaluation of the teeth and the third dimension can not be exactly assessed with conventional radiographic techniques including shifting the X-ray beam, some anatomic characteristics of the teeth may not be diagnosed.^5 , 6^ On the other hand, previous studies have shown that the root canal anatomy of all teeth including third molars is often extremely complex and highly variable.^7 - 9^

These anatomic variations may be a result of ethnic background, age, and gender.^10 - 12^ Few studies have focused on assessing the configurations of root canal anatomy and morphology in third molars. The purpose of current study was to investigate the root and canal morphology of mandibular third molars in an Iranian population.

## Materials and Methods

**Figure 1:**
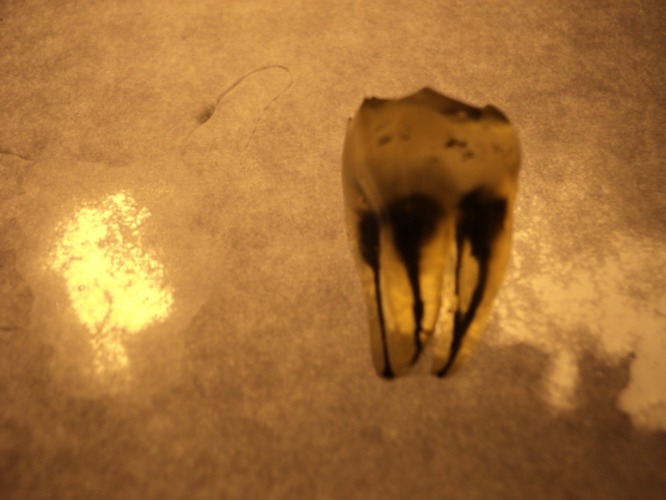
Photograph of a cleared mandibular third molar (×3 magnification).

**Figure 2:**
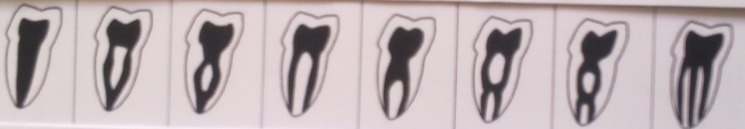
Types I–VIII (from left to right) of Vertucci’s classification for root canal anatomy of teeth.

One hundred fifty extracted mandibular third molars with completely formed apices were randomly collected from various dental clinics in Kerman, a province in southeast of Iran. Age, gender, and race of patients were not considered as criteria in this investigation. All attached soft tissue and calculus were removed using an ultrasonic scaler, subdivided according to the number of the roots, and soaked in 5.25% sodium hypochlorite (Shimin, Iran). Access cavities were prepared with a high speed hand piece and pulp tissue was dissolved by immersing the teeth in 5.25% sodium hypochlorite for 12 hours. Teeth were washed under running tap water for one hour and dried in room temperature over night. The location of apical foramina was established by passing a size 08 K-file (Maillefer, Swiss) into the canal until it penetrated the root apex. The India ink (Merck, Germany) was injected into the pulp chamber with an irrigating syringe with a 27-G needle. The ink was drawn through the canal system by applying negative pressure to the apical end of the tooth with the use of a central suction system. The stained teeth were air dried and demineralized with 6% nitric acid solution (Merck-Germany) for 5 days. The acid solution was changed daily and enough demineralization of the teeth was checked radiographically. Then, the teeth were washed and air dried. The samples were then dehydrated in ascending concentrations of Ethanol (Taghtir, Iran) for 12 hours and finally all specimens became transparent by immersion inside methyl salicylate solution (Merck, Germany). The teeth were kept inside this solution until becoming completely transparent. All stained and cleared samples were carefully examined under stereomicroscope (Olympus, Japan) using ×2 to ×3 magnifications (Figure 1). The number the type of the canals inside each root was recorded according to Vertucci classification (Figure 2).

## Results

From the total of 150 mandibular third molars studied, 21% had one root. The majority of teeth (73%) had two roots. 5.5% of the teeth had three roots. The incidence of c-shaped canal was 3.5% in this study and 8% of teeth had at least one dilacerated root. The results of current study are summarized in the Table 1. The number and the type of the canals in each root according to the Vertucci classification are shown in Tables 2, 3, and 4. All four canals of the only four-rooted tooth studied were of type I.

**Table 1:** Number of canals and roots in mandibular third molars of the studied population (n=150)

	Number of roots			
Teeth (n)	1	2	3	4
1	10	—	—	—
2	16	59	—	—
3	4	42	7	—
4	—	5	1	1
C-shaped	2	3	—	—

**Table 2:** Classification of root canals according to Vertucci classification in the studied single-rooted mandibular third molars

Teeth (n)	Type of canal
10	Type I
7	Type II
3	Type III
8	Type IV
1	Type V
1	Type VIII
2	C-shaped

**Table 3:** Classification of root canals according to Vertucci’s classification in the studied two-rooted mandibular third molars

Teeth (n)	Mesial root	Distal root
59	Type I	Type I
19	Type II	Type I
15	Type III	Type I
2	Type IV	Type I
6	Type V	Type I
3	Type IV	Type III
2	Type V	Type II
3	C-shaped

**Table 4:** Classification of root canals according to Vertucci’s classification in the studied three-rooted mandibular third molars

Teeth (n)	Mesiobacal root	Mesiolingual root	Distal root
5	Type I	Type I	Type I
1	Type I	Calcified	Type I
1	Type I	Calcified	Type II
1	Type I	Type I	Type II

## Discussion

Although several techniques have been used by different investigators in order to clarify the root canal anatomy and morphology of the teeth, it has been stated that clearing the stained root canals is the best method to provide a three-dimensional image from the root canal systems of the teeth.^13^ Some authors believe that spiral computed tomography (SCT), which is mainly being used in treatment planning for dental implants, is superior to staining and clearing method in root canal anatomy and morphology studies.^14 - 18^ In the case of clinical situations and in vivo studies, SCT is an excellent method for observing the buccolingual dimension of the teeth and related anatomic landmarks. However, it may not be possible to utilize this novel technology for the evaluation of microscopic lateral and accessory canals as well as other important features like intercanal communications, delta or loops. India ink is partly able to penetrate fine semicalcified canals or at least stain their orifices, helping to detect them.^19^ By applying the clearing technique, we found 1 to 4 roots for mandibular third molars and the number of canals ranged from 1 to 4 inside these roots. We did not observe any mandibular third molars having 5 or 6 root canals. The results of this study is similar to previous findings also reporting 1 to 4 roots for mandibular third molars using clearing technique.^20^ Only the latter study has evaluated root canal anatomy and morphology of mandibular third molars in an acceptable number of extracted teeth, while other papers are just case reports discussing a special anatomic variety of one third molar tooth.

Comparison the results of current study with those of the latter study reaches to this conclusion that having two roots is the normal anatomy for mandibular third molars, since the range of 73% obtained from our study is very similar to that of Sidow & West (77%).^20^ In the current study, 8% of teeth had at least one dilacerated root, which is a high prevalence in comparison with other teeth of the dentition. This may be due to the late eruption of third molar teeth, so that the related eruption space in many cases has already been filled by other teeth of the dentition. To the best of our knowledge, this is the first study which classifies the morphology of mandibular third molar roots according to Vertucci’s classification.^21^

## References

[1] hargreaves km, cohen s (2011). pathways of the pulp.

[2] olson dj, roberts s (2008). unevenness of the apical construction in human maxillary central incisors. j endod.

[3] baratto f, zaitter s (2009). analysis of the internal anatomy of maxillary first molars by using different methods. j endod.

[4] martos j, lubian c (2010). morphologic analysis of the root apex in human teeth. j endod.

[5] olson ak, goerig ac (1991). the ability of radiograph to determine the location of the apical foramen. int endod j.

[6] jafarzadeh h, wu ny (2007). the c-shaped root canal configuration: a review. j endod.

[7] rahimi s, shahi s, lotfi m, zand v (2008). root canal configuration and the prevalence of c-shaped canals in mandibular second molars in an iranian population. j oral sci.

[8] walton r, torabinejad m (1996). principles and practice of endodontics.

[9] ash m, nelson s (2003). wheeler’s dental anatomy physiology and occlusion.

[10] wein fs, hayami s (1999). canal configuration of the mesiobuccal root of the maxillary first molars of a japanese sub-population. int endod j.

[11] neaverth ej, kuttler lm (1987). clinical investigation (in vivo) of endodontically treated maxillary first molars. j endod.

[12] fogel h, peikof md (1994). canal configuration in the mesiobuccal root of the maxillary first molar: a clinical study. j endod.

[13] ahmed ha, abubakr na (2007). root and canal morphology of permanent mandibular molars in a sudanese population. int endod j.

[14] reuben j, velmurugan n (2008). the evaluation of root canal morphology of the mandibular first molars in an indian population using spiral computed tomography scan: an in vitro study. j endod.

[15] neelakantan p, subbarao c, subbarao cv (2010). comparative evaluation of modified canal staining and clearing technique, cone-beam computed tomography, peripheral quantitative computed tomography, spiral computed tomography and plain and contrast medium-enhanced digital radiography in studying root canal morphology. j endod.

[16] deepalakshmi m, miglani r, indira r, ramachandran s (2010). spiral ct diagnosis and endodontic management of an anatomically variant palatal root with two canals in a maxillary first molar. indian j dent res.

[17] meder c, cowherd l, williamson ae, johnson wt (2011). apical morphology of the palatal roots of maxillary molars by using micro-computed tomography. j endod.

[18] yamada m, ide y, matsunaga s, kato h (2011). three dimensional analysis of mesiobuccal root canal of japanese maxillary first molar using micro-ct. bull tokyo dent coll.

[19] kuzekanani m, asgary e (2005). the incidence of mandibular first premolar teeth with 2 canals in a group of kerman population. beheshti university dental journal.

[20] sidow sj, west la (2000). root canal morphology of human maxillary and mandibular third molars. j endod.

[21] vertucci fj (1984). root canal anatomy of the human permanent teeth. oral surg oral med oral pathol.

